# Subclinical growth of an arteriovenous fistula associated with renal biopsy: a case report

**DOI:** 10.1186/s12882-016-0289-4

**Published:** 2016-07-12

**Authors:** Takuya Murakami, Shin-ichi Takeda, Hidenori Kanazawa, Atsushi Ugajin, Shigeyoshi Kijima, Hiroyasu Nakamura, Toshimi Imai, Taro Sugase, Ryoko Horikoshi, Takahisa Kobayashi, Tetsu Akimoto, Osamu Saito, Daisuke Nagata

**Affiliations:** Division of Nephrology, Department of Medicine, Jichi Medical University, 3311-1 Yakushiji, Shimotsuke, Tochigi Japan; Department of Radiology, Jichi Medical University, 3311-1 Yakushiji, Shimotsuke, Tochigi Japan

**Keywords:** Renal biopsy, Renal arteriovenous fistula, Interventional radiology

## Abstract

**Background:**

Renal biopsy is not free from complications and patients who undergo this procedure are usually hospitalized to receive intensive care for several days after biopsy. In contrast, after this period, routine follow-up to detect biopsy-associated complications is rarely scheduled, unless the patient develops a clinical manifestation. We describe a case of marked enlargement of arteriovenous fistula in the kidney that occurred many years after renal biopsy. In contrast to the previous cases requiring interventional radiology, our patient showed subclinical growth of fistula over about nine years.

**Case presentation:**

A 24-year-old man with a history of percutaneous renal biopsy was hospitalized for interventional radiology. Gross hematuria emerged shortly after biopsy, but completely disappeared with administration of hemostatic agents and bed rest. Subsequently, the patient had few symptoms for many years. A giant fistula (a gourd-shaped mass, size 26 × 22 and 12 × 11 mm) was unexpectedly detected by ultrasonography performed for examination of an unrelated disorder (slight elevation of serum transaminase) at 9 years after the original biopsy. The fistula was successfully treated with radiological intervention. Thus, subclinical development of complications associated with renal biopsy should be considered, even in an uneventful course.

**Conclusions:**

This case provides a platform to discuss the importance of long-term follow-up of patients after renal biopsy despite of its difficulty.

## Background

Renal biopsies provide critical clues in diagnosis of renal diseases. However, this procedure is not free from complications, despite technical improvements over time. A recent nationwide study in Norway showed gross hematuria after biopsy in 1.9 % of patients, with 0.9 % needing blood transfusion and 0.2 % requiring surgical intervention or catheterization [1]. There have also been several reports of renal arteriovenous fistula (AVF) after renal biopsy. In most such cases, radiological interventions were performed due to severe clinical manifestations of gross hematuria [2–4], hemorrhagic shock [2], severe hypertension [2, 5], and decline of renal function [4], which all emerged shortly after the biopsy. We herein report a case of marked enlargement of renal AVF that was detected by chance nine years after biopsy and was successfully treated with interventional radiology (IR). In contrast to the previous cases of renal AVF associated with biopsy [2–6], our patient showed subclinical growth of AVF over about nine years. Thus, this case provides a platform to discuss the importance of long-term follow-up of patients after renal biopsy.

## Case presentation

**Fig. 1:**
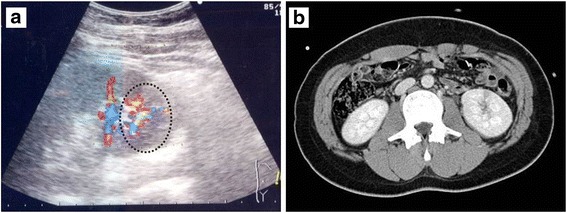
Initial appearance of the AVF. **a** Color-coded Doppler US shortly after biopsy showed the AVF as a mosaic signal in the lower pole of the left kidney. **b** There were morphologically few abnormalities in the vasculature on dynamic contrast-enhanced CT, indicating the small size of this initial lesion

A 24-year-old man was admitted to our facility for IR. The patient had a history of percutaneous kidney biopsy under real-time ultrasound guidance using a cutting needle at 15 years of age. Macroscopic hematuria started shortly after biopsy, but ceased within a couple of days without development of significant anemia. The size of a subcapsular hematoma (up to 38 mm long by 7 mm wide), which occurs in most renal biopsies, also decreased over time. Thus, common complications such as gross hematuria and subcapsular hematoma occurred, but were settled within days of the biopsy. Based on our institutional protocol, the patient was discharged 7 days after biopsy. However, immediate rehospitalization was required on the day of discharge due to abrupt onset of severe left flank pain. Gross hematuria reemerged and was intermittently observed for several days, resulting in hypotension (94/58 mmHg), a significant decrease of blood hemoglobin (14.2 to 10.7 g/dl), and acute dysuria. Embolization via IR was considered as an emergency therapy to stop bleeding, but gross hematuria eventually disappeared within days with intravenous injection of hemostatic agents such as carbazochrome sodium sulfonate and tranexamic acid, in addition to bed rest. It was noteworthy that color-coded Doppler US detected AVF as a mosaic signal in the lower pole of the left kidney (Fig. 1a), which was the puncture site of the biopsy. However, in terms of size, this lesion was morphologically undetectable, even by dynamic contrast-enhanced computed tomography (CT) (Fig. 1b). After disappearance of gross hematuria, the patient was free from renal AVF-related manifestations, including abdominal pain, hypertension, and renal impairment. Thus, it was determined that IR was not required at this time.

IgA nephropathy was diagnosed based on the findings of renal biopsy. The patient was hospitalized again six months later to receive steroid pulse therapy followed by tonsillectomy [7] for this glomerulopathy. A striking improvement in urinary abnormalities was gradually achieved, with urinary protein reduced from 4.2 to 0.2 g/day and red blood cells decreased to a level of 5 cells per high-power field in sediment. Ultrasound examination was performed again, but growth of renal AVF was not evident in this period. Following the combination therapy, the patient regularly visited our hospital and took 300 mg dipyridamole and 6 mg candesartan orally each day. The angiotensin II receptor antagonist was prescribed as a renoprotective agent, rather than for a depressor effect. Blood pressure (125/75 mmHg), blood hemoglobin, and serum creatinine remained normal in this period and for over eight years.

**Fig. 2:**
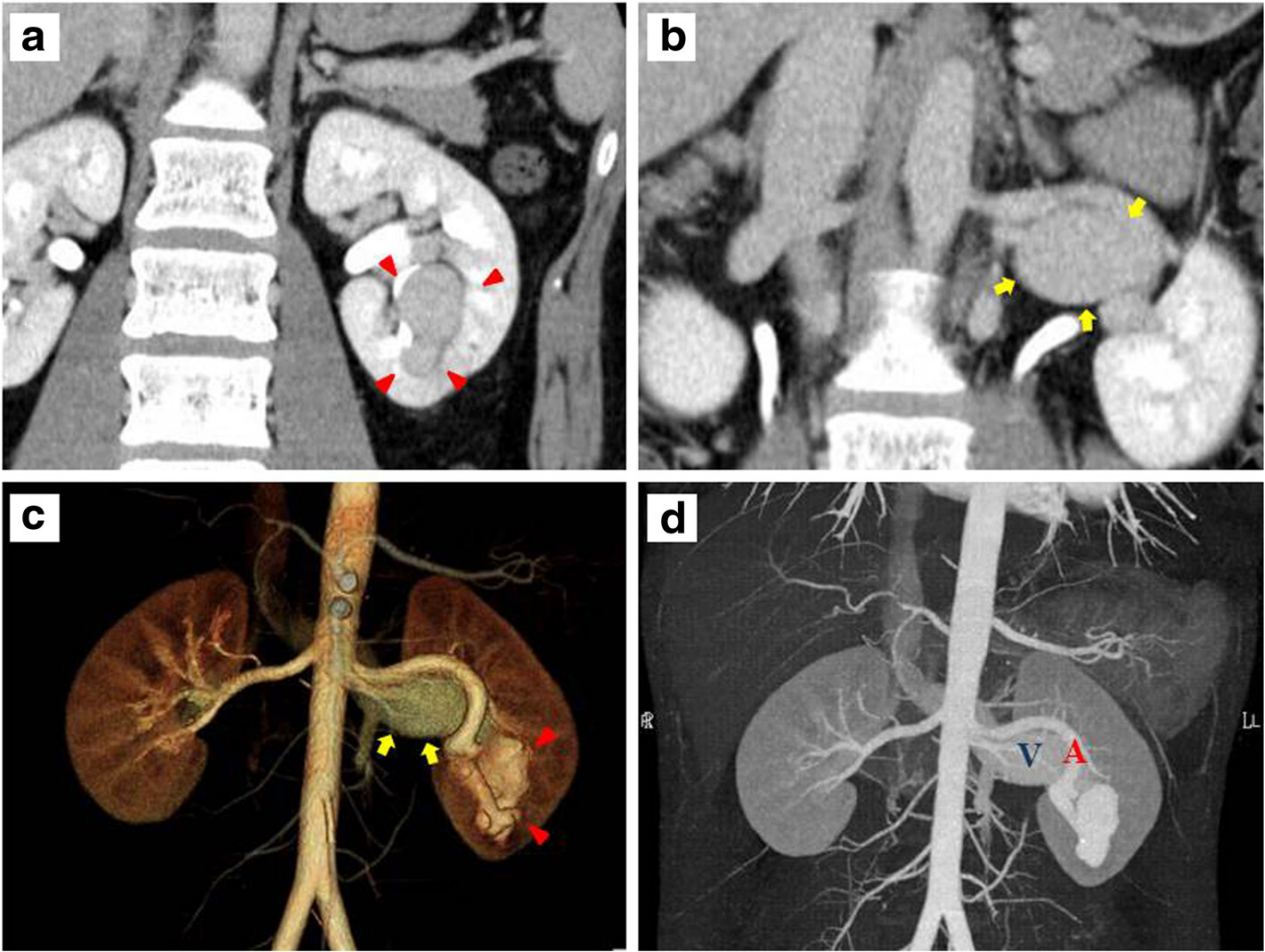
Contrast-enhanced CT of the enlarged AVF and dilatation of the left renal vein. Two-dimensional (**a**, **b**) and three-dimensional (**c**) CT performed incidentally at 9 years after biopsy revealed the morphological characteristics of an enlarged left renal AVF (*red arrowheads*) and dilated left renal vein (*yellow arrows*). **d** A maximum intensity projection image showed a connection with the renal artery and vein. Abbreviations: A, renal artery; V, renal vein

Despite the uneventful course, at the age of 24 the patient underwent abdominal ultrasound because of a slight elevation of serum alanine aminotransferase (up to 36 U/l), which had been noted approximately one year earlier. Unexpectedly, a mass lesion was found in the left kidney, whereas there were few morphological abnormalities in the liver. Dynamic contrast-enhanced CT subsequently delineated a gourd-shaped mass (26 × 22 and 12 × 11 mm) in the left kidney (Fig. 2a) and marked dilatation of the left renal vein (Fig. 2b). Three-dimensional CT (Fig. 2c) and maximum intensity projection CT (Fig. 2d) clearly revealed that this lesion directly led to the renal artery and vein, suggesting that the renal AVF had grown subclinically to a giant size over many years and had resulted in marked dilatation of the venous system. Therefore, IR was required to prevent further growth of the fistula and manifestation of symptoms.

**Fig. 3:**
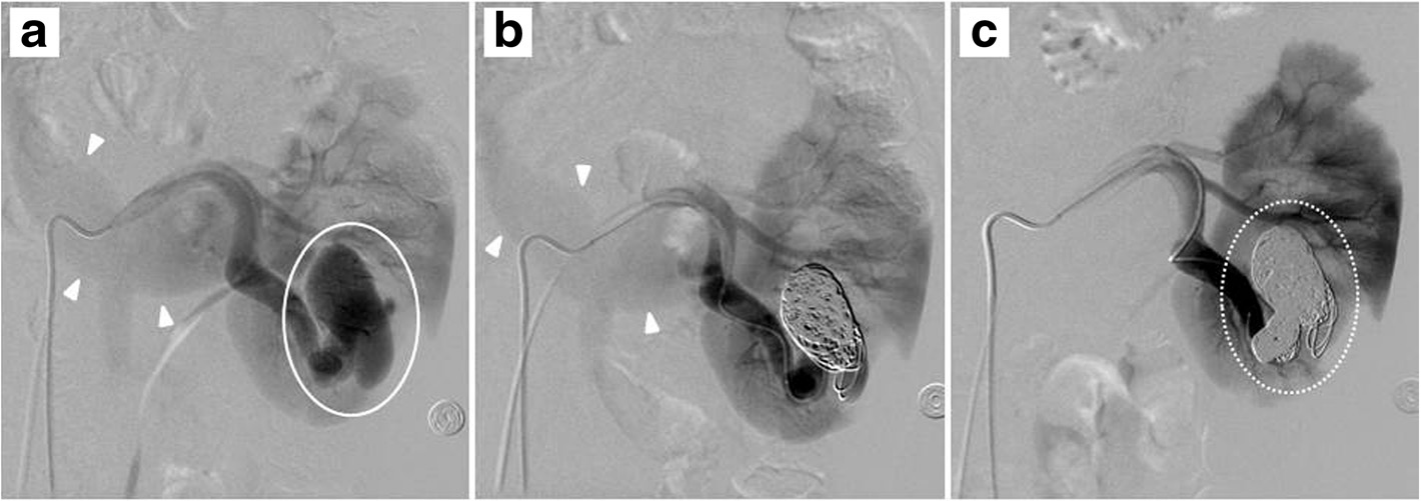
Embolization therapy for the renal AVF. **a** Pre-therapeutic, **b** early treatment phase, and **c** post-therapeutic digital subtraction angiography images. The renal AVF (*circled with a solid line*) was filled with microcoils (*circled with a dashed line*) over time. The enlarged left renal vein (indicated by *arrowheads*) was delineated despite the arterial phase until the early therapeutic period (**a**, **b**), but disappeared after the AVF was blocked out (**c**), indicating a large amount of shunt flow

**Fig. 4:**
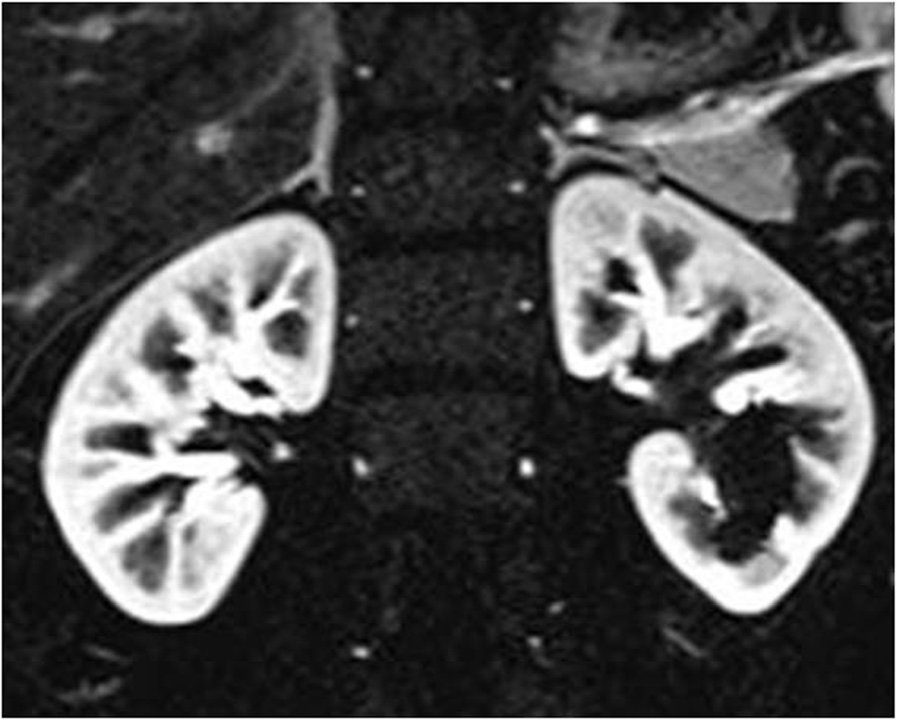
Follow-up study after IR. Contrast-enhanced MRI demonstrated the success of IR treatment, which resulted in discontinuation of blood flow to the AVF with preservation of renal blood flow

Transcatheter embolization of high-flow left renal AVF was performed using detachable coils by radiologists. The fistula was solidly packed with 7 Coil 400 Standard Complex® coils (Penumbra, Inc., Alameda, CA, USA) and 22 Presidio® coils (Codman, Inc., Raynham, MA, USA) under digital subtraction angiography. As shown in Fig. 3, the high-flow fistula was successfully blocked out, with conservation of renal blood flow. The patient was discharged on postoperative day 3 without adverse events. A follow-up test by contrast-enhanced MRI at about 3 months after IR showed maintained discontinuation of blood flow to the AVF (Fig. 4), indicating the success of the treatment.

## Conclusions

Complications associated with renal biopsies are not uncommon. Indeed, nephrologists exert extreme caution early after puncture, especially for the first few days. The guidelines for renal biopsies published by the Japanese Society of Nephrology [8] recommend a stay of no less than 4 to 7 days in hospital. Postural changes in bed and adoption of a standing position are not allowed for the first hours and almost the entire first day, respectively, based on two studies of biopsy-related complications: Khajehdehi et al. [9] found that patients with stable hematocrit at 6 h were at low risk for bleeding at 24 h while hospitalized, and Marwah et al. [10] found that major complications were identifiable in most cases within 12 h. Thus, intensive management is important for a short time after renal biopsy. However, there is little information on appropriate follow-up for patients with a history of renal biopsy after this period.

A number of cases of renal AVF associated with biopsy [2–6] have been published. In particular, Lorenzen et al. [6] reported 20 cases of post-biopsy AVF, all of which were successfully treated with IR. In this report, the incidence of AVF after renal biopsy was 1.6 %, but this rate is as high as 16 % in other reports. Lorenzen et al. also highlighted the improvement of renal function after IR, based on a significant decrease in the mean creatinine level from 4.4 to 2.7 mg/dl (*p* = 0.0014). In our case, IR was initially considered because of gross hematuria, but was not used in light of the subsequent uneventful course. Since 70 % of AVFs occlude spontaneously, medical management in anticipation of spontaneous closure of the fistula may be the best first choice in an asymptomatic patient [6]. In most other IR cases, patients with renal AVF showed severe clinical manifestations including gross hematuria [2–4], hemorrhagic shock [2], severe hypertension [2, 5], and decline of renal function [4].

The present case raises an issue regarding long-term follow-up of patients after renal biopsy. This may not be feasible in practice for all patients and some might not need regular visits, depending on the diagnosis. However, our case shows the possibility of subclinical development of renal biopsy-associated complications indicating a subpopulation of patients that requires periodic examination, even if this is uneventful. Based on our experience, this subpopulation may include patients who are younger and who show clinical manifestations shortly after biopsy.

In conclusion, we have reported a case of marked enlargement of a renal AVF that was found incidentally 9 years after biopsy without clinical manifestations and was successfully treated with IR.

## Abbreviations

AVF, arteriovenous fistula; CT, computed tomography; IR, interventional radiology; US, ultrasonography
